# Forensic differentiation between peripheral and menstrual blood in cases of alleged sexual assault—validating an immunochromatographic multiplex assay for simultaneous detection of human hemoglobin and D-dimer

**DOI:** 10.1007/s00414-017-1719-y

**Published:** 2017-10-23

**Authors:** Hannah Holtkötter, Claudemir Rodrigues Dias Filho, Kristina Schwender, Christian Stadler, Marielle Vennemann, Ana Claudia Pacheco, Gabriela Roca

**Affiliations:** 1 Institute of Legal Medicine, University of Münster, Röntgenstraße 23, 48149 Münster, Germany; 2 Superintendência de Polícia Técnico-Científica, Instituto de Criminalística, São Paulo, Brazil; 3 SERATEC Gesellschaft für Biotechnologie mbH, Ernst-Ruhstrat-Straße 5, 37079 Göttingen, Germany

**Keywords:** Body fluid identification, Menstrual blood, D-dimer, Immunochromatographic assay, Sexual assault

## Abstract

Sexual assault is a serious offense and identification of body fluids originating from sexual activity has been a crucial aspect of forensic investigations for a long time. While reliable tests for the detection of semen and saliva have been successfully implemented into forensic laboratories, the detection of other body fluids, such as vaginal or menstrual fluid, is more challenging. Especially, the discrimination between peripheral and menstrual blood can be highly relevant for police investigations because it provides potential evidence regarding the issue of consent. We report the forensic validation of an immunochromatographic test that allows for such discrimination in forensic stains, the SERATEC PMB test, and its performance on real casework samples. The PMB test is a duplex test combining human hemoglobin and D-dimer detection and was developed for the identification of blood and menstrual fluid, both at the crime scene and in the laboratory. The results of this study showed that the duplex D-dimer/hemoglobin assay reliably detects the presence of human hemoglobin and identifies samples containing menstrual fluid by detecting the presence of D-dimers. The method distinguished between menstrual and peripheral blood in a swab from a historical artifact and in real casework samples of alleged sexual assaults. Results show that the development of the new duplex test is a substantial progress towards analyzing and interpreting evidence from sexual assault cases.

**Electronic supplementary material:**

The online version of this article (10.1007/s00414-017-1719-y) contains supplementary material, which is available to authorized users.

## Introduction

Identifying the biological source of a crime scene stain is one of the most important components in forensic science practice. This aids police investigations as it possibly provides the investigators with information on the course of the crime. Blood is one of the most commonly found body fluids at crime scenes, and accurate differentiation between peripheral blood and menstrual fluid could provide crucial evidence, e.g., regarding the issue of consent in sexual assault cases; while the presence of peripheral blood indicates a traumatic cause, menstrual fluid points towards a natural bleeding cause [1]. Some body fluids present in cases of alleged sexual assault, such as semen or saliva [2, 3], can be detected reliably, but others are more challenging to detect, such as pre-ejaculate, menstrual blood, or vaginal fluid. Driven by the importance of forensic body fluid identification, novel methods have been extensively researched over the years including microscopic examination [4, 5], messengerRNA (mRNA) [6, 7], microRNA (miRNA) [8, 9], and DNA methylation profiling [10–12], which have been successfully applied to the identification of blood. However, distinguishing peripheral blood from menstrual fluid with these methods has been shown to be highly challenging, and the application of more complex molecular techniques requires a considerable amount of training and experience. Furthermore, results may be influenced by inter- and intra-individual differences between donors [13], and a full forensic validation for casework application of these methods is hitherto still outstanding. The most important requirements of newly developed tests are that they do not interfere with or hamper subsequent DNA analysis by loss of material, contamination, or inhibition.

Immunochromatographic assays for body fluid detection have been shown to be highly specific and easy to use. Recently, initial work successfully introduced immunochromatographic assays that detect degradation products of fibrinolysis (FDPs) as innovative methods for the identification of menstrual fluid in forensic samples; during menstruation fibrinolysis, the endogenous degradation of fibrin after blood coagulation is a crucial step that enables menstrual fluid to easily pour out [1, 15]. The most significant subtype of FDPs is D-dimer, a small protein fragment specific to the process of fibrinolysis. The use of D-dimers for menstrual fluid identification has first been described by Miyaishi et al. in 1996 [14] who showed that menstrual blood has a mean concentration of D-dimer 200 times greater than that of peripheral blood. With that in mind, Baker and colleagues [15] examined four D-dimer assays, examining their specificity and sensitivity in detecting menstrual blood. Results showed that Clearview® Simplify D-Dimer (Alere, Cheshire, UK) was the kit expressing highest sensitivity and specificity, beyond which, its usage was simple and quick, and results were easy to interpret. The kit, originally developed for diagnostic purposes to detect D-dimers in peripheral blood as a marker for peripheral thromboembolism, was validated in 2014 by our group for the detection of menstrual fluid in forensically relevant samples [1].

Recently, a novel immunochromatographic assay, the SERATEC PMB test (SERATEC GmbH, Göttingen, Germany), was developed, which combines the detection of human hemoglobin and D-dimers. This is the first D-dimer assay that was developed specifically for differentiation between human peripheral blood and human menstrual fluid in forensic samples. To our knowledge, it also represents the first immunochromatographic multiplex assay for forensic use. The immunochromatographic SERATEC HemDirect test, which detects the presence of human hemoglobin in a forensic sample [16] and therefore confirms the presence of blood, was used as the basis for the development of the novel duplex test. According to SERATEC’s user instruction, the PMB assay detects hemoglobin down to a concentration of 20 ng/ml and D-dimer down to 400 ng/ml, is easy to use directly at crime scenes or in the laboratory, quick, and does not require special training of the analysts. Furthermore, the sample material remains suitable for DNA extraction and profiling [17].

Here, we report the final development and the forensic validation of the SERATEC PMB test. Different antibody quantities as well as buffer amounts and incubation times were tested. Moreover, we aimed to evaluate (1) the sensitivity of the PMB test by considering serial dilutions of peripheral blood and menstrual fluid, (2) possible influences of and cross-reactivity with other biological secretions, and blood from various species, (3) possible false-positive results on blood from aged donors and deceased’s peripheral blood, (4) the viability of DNA extraction and amplification from the residual blood diluted on the remaining buffer, (5) a case example involving a historical ethnological artifact, and (6) the performance of the test on blood samples from ten cases of alleged sexual assault.

## Material and method

### Test evaluation

The hemoglobin/D-dimer assay contains four monoclonal antibodies as active compounds and two polyclonal: two gold-labeled monoclonal for the respective epitopes of human hemoglobin and D-dimer located in the start area and two monoclonal antibodies for the sandwich assay located at the result lines. The two polyclonal antibodies are forming the control line. The cassettes of the assay contain a sample well and a result window with a control line (labeled “C” for control), a result line for hemoglobin (labeled “P” for peripheral blood), as well as a result line for D-dimer (labeled “M” for menstrual fluid). If no line is visible, the test is invalid and should be repeated [17].

Initially, five different cut-off levels for D-dimer detection were evaluated during production. Preliminary testing revealed that cut-off levels of ≥ 300 and ≥ 400 ng/ml were optimal for reliable detection of menstrual blood and avoidance of false-positive results from peripheral blood (data available on request). Two independent research institutions further evaluated these two cut-off levels.

### Sample collection

For the assessment of both cut-off levels and forensic validation of the assay, menstrual blood was collected on vaginal swabs from 16 healthy female donors (age ranging from 24 to 39 years old) at days 1, 2, and 3 of the menses. Informed consent was obtained from all individual participants included in the study. Liquid menstrual fluid was collected from one donor using a menstrual cup (Mooncup Ltd., Brighton, UK). Peripheral blood samples were collected from 14 healthy donors (six female and eight male). Swabs were stored at room temperature (RT) and liquid blood was stored frozen in aliquots, both for 8 months prior to testing. From dried samples, approximately 3 mm^2^ of the cotton swabs were used for testing. From liquid samples, 3 μl was used for testing and frozen samples were brought to RT prior to analysis.

### Forensic validation

The sensitivity of the PMB test was assessed by testing various volumes and dilutions of buffer and menstrual fluid: 3 μl of neat menstrual fluid were added to each 500, 1000, 1500, 3000, and 6000 μl of extraction buffer. Incubation times of 5, 30, 90, and 120 min were tested. Based on the results of the sensitivity, testing samples were prepared using the following protocols: the positive control was prepared with 3 μl menstrual fluid in 1000 μl buffer and extracted for 5 min. Fresh liquid samples were extracted in 1500 μl buffer for 5 min. Dry samples on cotton swabs were extracted in 300–500 μl buffer for 30 to 90 min. Aged-dried samples were extracted in 120 μl buffer for and extended incubation time of 120 min. Either 1/3, 1/2, or a full cotton swab was used for extraction, according to the amount of menstrual fluid deposited. After the individual incubation times 120 μl of sample-buffer solution was added to the sample well and results were read after a development period of 10 min.

The possibility of obtaining false-positive results was investigated using neat peripheral blood, vaginal fluid, semen, saliva, and urine as well as non-human peripheral blood samples from horse, cat, dog, goat, sheep, and rabbit. Mixtures of menstrual fluid were prepared with peripheral blood, vaginal fluid, semen, saliva, and urine (1:1).

In addition to the validation samples, postmortem samples were tested for possible false-positive results due to potential postmortem fibrinolysis [14, 18]. For this, peripheral blood from deceased (*n* = 10) was applied to cotton swabs during medicolegal autopsy and air-dried at RT for 4 weeks prior to testing. As D-dimer levels are known to increase with age [19], five peripheral blood samples obtained from donors over the age of 60 years were collected with informed consent. The samples were tested by adding 3 μl of blood to 1500 μl of buffer, and additionally by adding 1 μl of blood to a cotton swab that was extracted in 120 μl of buffer after a storage time of 2 weeks.

All samples were tested in duplicates and the provided buffer was used as negative control.

### DNA analysis

DNA profiles were generated from a mixture of menstrual fluid and semen. DNA was extracted from (1) the pad underneath the sample well and (2) the remaining sample-buffer solution. For method 1, the plastic cartridge was opened and the pad was cut out and transferred to a reaction tube. For method 2, 100 μl of sample-buffer solution was transferred to a reaction tube. DNA was extracted using the Maxwell® instrument (Promega, Mannheim, Germany) and amplified with PowerPlex® ESX 17 Pro System (Promega) according to manufacturer’s recommendations. Samples were subsequently analyzed using the 3130 Genetic Analyzer with the GeneMapper® ID software by Thermo Fisher Scientific.

### Ethnological artifact case example

A case study was performed that involved a historical artifact, an African statue used during traditional ceremonies for medicinal purposes and administration of justice in a rural community of Gabon. The statue of unknown age was made from wood and was suspected to be covered in human blood and/or menstrual fluid. A swab was taken from the statue and approximately 3 mm^2^ was submerged in 500 μl of the provided SERATEC PMB extraction buffer. After an incubation time of 120 min 120 μl was applied to the sample well and results were read after a development period of 10 min. Also, the swab was analyzed using the human-specific RSID-Blood test (Galantos Genetics, Mainz, Germany) for validation.

### Sexual assault case samples

Lastly, blood samples from ten cases of alleged sexual assault were analyzed using the SERATEC PMB test. Blood samples were either collected from the victims’ clothing or from vaginal swabs collected during the medical examination. The existence of human blood was additionally tested using the Feca-Cult One Step Test (Alamar Tecno Científica Ltda), an immunochromatographic test for the qualitative determination of human hemoglobin. All tests were performed in duplicates. The volume of the provided SERATEC PMB extraction buffer was 300 or 500 μl and the incubation time varied from 30 to 90 min, considering the amount of blood on each sample (see Table 1). The test dose was 120 μl applied to the sample well and results were read after a development period of 10 min. A brief history of each case and sample is given in the supplementary material, document 1.

**Table 1:** Summary of the alleged sexual assault cases with information on samples, storage time, testing procedure, and results

Case #	Time since last menses	Vaginal lesions found during medical examination	Evidence	Sample appearance	Storage time	Material used	Buffer (μl)	Incubation time (min)	D-dimer presence	Hemoglobin presence
1	10 days	Yes	Panty	dark red	47 days	25 mm^2^	500	30	–	+
			Dress	dark red	47 days	25 mm^2^	500	30	–	–
2	unknown	Yes	Panty	light red	57 days	25 mm^2^	300	30	–	+
3	unknown	unknown	Panty	dark red	159 days	25 mm^2^	500	30	+	+
4	3 days	No	Vaginal swab	light red	514 days	1/3 swab	300	90	+	+
5	8 days	No	Vaginal swab	light red	497 days	1/3 swab	300	90	–	+
6	10 days	No	Vaginal swab	light red	507 days	1/3 swab	300	90	–	+
7	10 days	No	Vaginal swab	light red	537 days	1/3 swab	300	90	+	+
8	2 days	No	Vaginal swab	light red	491 days	1/3 swab	300	90	+	+
9	1 day	No	Vaginal swab	light red	500 days	1/3 swab	300	90	+	+
10	1 day	No	Vaginal swab	light red	429 days	1/3 swab	300	90	+	+

## Results and discussion

### Test evaluation

**Fig. 1:**
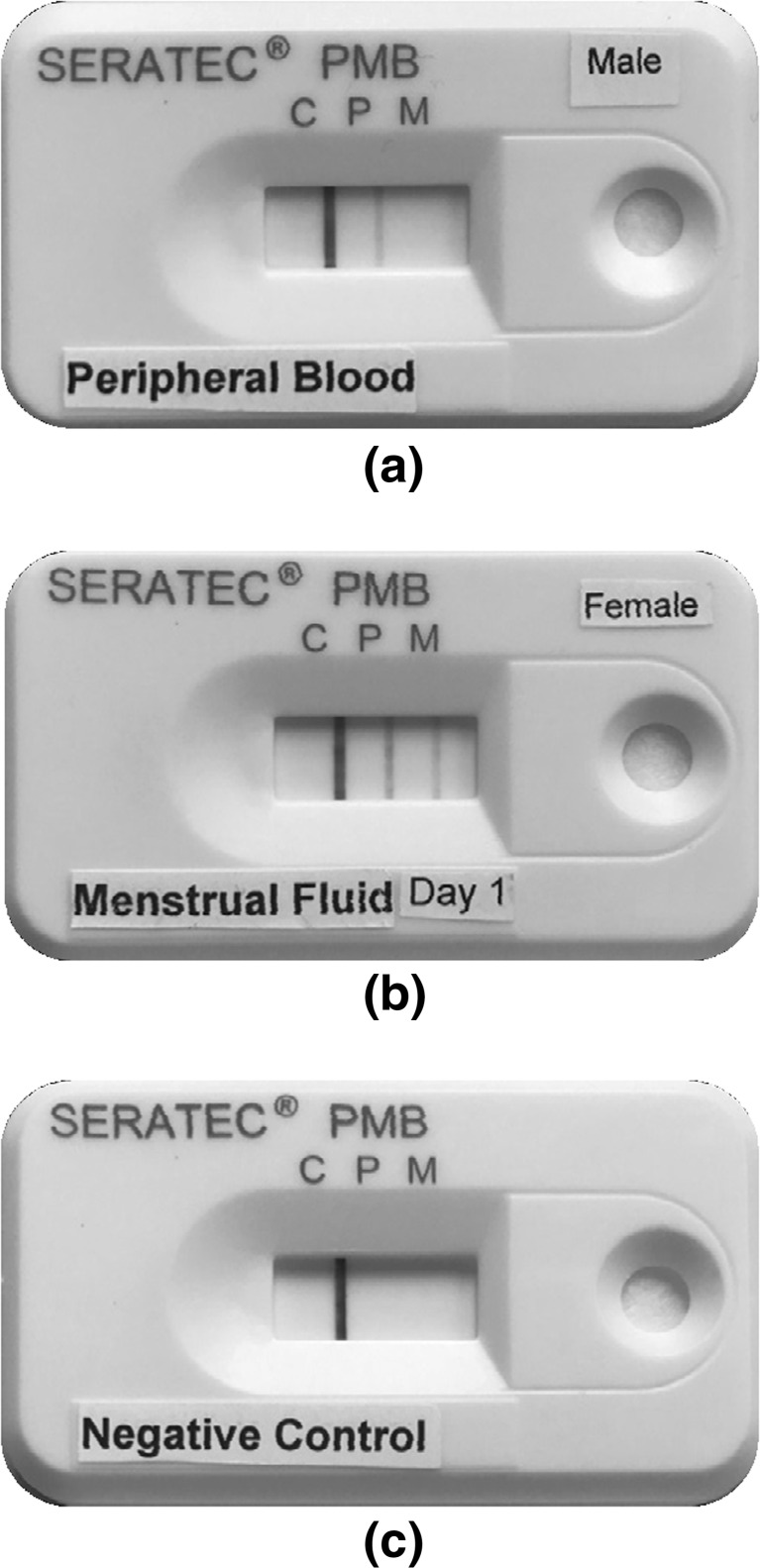
Examples for a positive peripheral blood test (**a**) and positive menstrual fluid test (**b**). The peripheral blood test shows a positive hemoglobin (P) signal and no false-positive D-dimer (M) signal. Menstrual blood reacts positive for hemoglobin (P) and D-dimer (M). The negative control (**c**) shows no signal for hemoglobin or D-Dimer. A signal for correct test performance (C) is present in all three examples

Handling of the test device (see Fig. 1 for examples of the cassettes) was easy and straightforward. All replicates showed consistent results and all negative controls gave the expected result (i.e., line “C” present, lines “P” and “M” not).

**Fig. 2:**
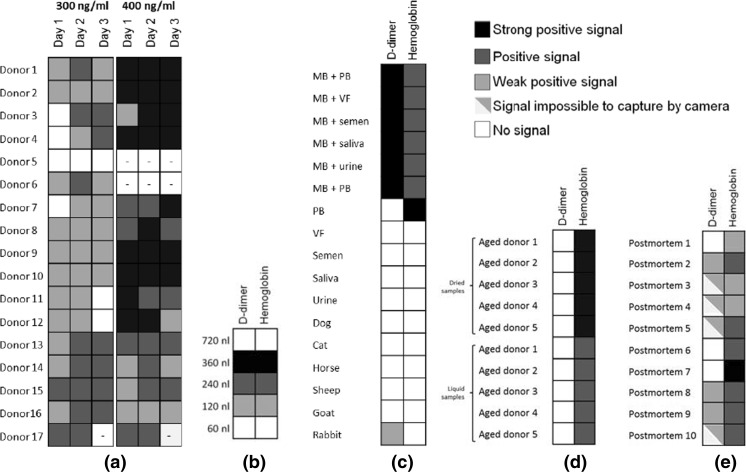
Heat maps of **a** signals for D-dimer presence with the 300 and 400 ng/ml cut-off (donor 1 to 16 = dried blood, donor 17 = liquid blood), **b** the sensitivity study, **c** the mixtures and cross-reactivity testing, **d** blood collected from aged donors, and **e** postmortem blood samples. *MB* menstrual blood, *PB* peripheral blood, *VF* vaginal fluid

All tests with 300 ng/ml as well as 400 ng/ml as cut-off for the D-dimer antibody worked correctly as shown by the test’s C line. All tests reacted positive for hemoglobin presence and negative for D-dimer presence when peripheral blood was tested. Furthermore, all of the menstrual blood samples gave a positive result for hemoglobin presence, dried (*n* = 16) or liquid (*n* = 1). While fresh liquid menstrual fluid reacted clearly positive for D-dimer presence with both cut-offs, dried menstrual fluid samples showed more varied results (see Fig. 2a for details).

#### Results for the cut-off of 300 ng/ml using stored dry menstrual blood (*n* = 16)

From the 48 samples tested (16 donors, days 1, 2, 3), 40 reacted positive (83.3%) and eight reacted false negative (16.7%) to the presence of D-dimer. Only 14 of the samples that reacted positive gave a clear positive signal (29.2%) while the majority of the samples only gave a weak positive signal (54.1%). None of the samples gave a strong positive signal.

#### Results for the cut-off of 400 ng/ml using stored dry menstrual blood (*n* = 14)

All of the 42 samples tested (14 donors, days 1, 2, 3) reacted positive to the presence of D-dimer (100%). The majority gave a strong positive signal (52.4%), 12 from the 42 samples gave a clear positive reaction (28.6%), and only eight of the samples gave a weak positive signal (19%). It was observed that intra-individual differences of the samples result in variances of signal intensity. The day of the menses on which the sample was collected does not seem to influence the results of the test.

Based on these results, the following tests were performed using the 400 ng/ml cut-off only.

### Forensic validation

The sensitivity testing revealed that menstrual fluid was still detectable down to 120 nl, which is equivalent to 3 μl of menstrual fluid diluted in 3000 μl buffer. Here, a clear signal for hemoglobin and an extremely weak signal for D-dimer were observed. A much clearer signal for D-dimer presence was observed when only 1500 μl buffer was used on 3 μl of sample; thus, 240 nl of menstrual fluid. Therefore, the detection limit with the PMB test was set to 240 nl of menstrual fluid. Best results, with clear, balanced signals for both hemoglobin and D-dimer presence, were received with a concentration of 360 nl of menstrual fluid (3 μl in 1000 μl buffer), which is why this was set as optimal for a positive control. It was observed that 3 μl of fresh liquid menstrual fluid in only 500 μl of buffer (720 nl) overloaded the test strips and signals were hard to interpret. Hence, when fresh liquid samples are received for analysis, they should either be diluted beforehand or used with the appropriate amount of at least 1000 μl of buffer. No signal was received when 60 nl of menstrual fluid (3 μl in 6000 μl buffer) was analyzed.

Different incubation times were tested for liquid and dried samples. It was shown that for liquid samples, an incubation time of only 5 min is sufficient. For dried samples, individual decisions on incubation times have to be made by the analyst, depending on the amount of blood deposited on the evidence and the time of deposition. From experience in the application of this test, we suggest the following: a tampon or sanitary pad should generally contain an enormous amount of blood. If it is relatively freshly collected, it is advised that a piece of either is extracted in 500 μl for 30 min. If it has been stored for several weeks or months, the extraction time should be extended to 60 min. For smaller blood stains, i.e., collected on clothing, the amount of buffer should be reduced to 300 μl. If an object is swabbed or only traces of blood are found, the minimal amount of 120 μl buffer should be used. If the swab has been deposited for months or years before analysis, the incubation time should be extended to 90 min.

To assess whether the presence of other body fluids influence the test, mixtures and neat body fluids were analyzed. All of the mixtures containing menstrual fluid gave a clear positive result for D-dimer presence and no false positive was observed by peripheral blood, vaginal fluid, semen, saliva, and urine (Fig. 2c). To assess cross-reactivity with other species, animal blood samples were tested. Blood samples collected from dog, cat, horse, sheep, goat, and rabbit reacted negative for the presence of human hemoglobin. With the exception of rabbit blood, which reacted very weakly positive for the presence of D-dimer, all other animal blood samples gave a negative result for D-dimer. Since rabbit blood reacted negative for hemoglobin presence, and a positive reaction would be expected in case of a menstrual blood sample, this result does not pose a problem for casework samples. Positive D-dimer reaction combined with negative hemoglobin reaction might indicate a non-human origin of a suspected blood stain.

All samples from donors over the age of 60 (*n* = 5) tested positive for peripheral blood and negative for menstrual fluid (Fig. 2d). Slightly stronger signals were obtained for dried samples than for liquid samples. Thus, no false-positive D-dimer results were generated by these samples; hence, samples from elderly people do not seem to influence test results.

All of the postmortem samples (*n* = 10) reacted positive for hemoglobin presence and seven of the samples additionally reacted positive for D-dimer presence. Of the seven samples three gave a weak positive signal while four gave such a light signal that was impossible to be captured by camera (Fig. 2e). Numerous biochemical reactions take place in dying cells and corpses. The one that is likely to explain the positive reaction to D-dimer presence is a decrease in oxygen levels that leads to a switch to anaerobic metabolism. In turn, this results in the accumulation of lactic acid, initiating a fall in pH postmortem. The plasma pH decrease is noteworthy because it is believed to introduce fibrinolysis, and therefore, the formation of D-dimers [18, 20]. Consequently, care must be taken when postmortem blood samples need to be assessed.

### DNA analysis

It was possible to generate DNA profiles from the sample-buffer solution as well as from the pad beneath the sample well. The two STR profiles are given in the supplementary material, document 2. As a relatively high amount of the D-dimer incubation buffer is used for each sample, the possibility to subsequently perform standard methods such as DNA profiling from leftover buffer solution is particularly useful. This avoids sample loss for body fluid identification. The possibility to extract DNA from the pad is especially important in cases where the entire amount of sample-buffer solution was needed for analysis. No differences in peak height (rfu) were observed between the two extraction methods.

### Ethnological artifact case example

Using the SERATEC PMB test, the swab taken from the African figurine tested positive for the presence of hemoglobin and negative for the presence of D-dimer. With the RSID-Blood test, the sample tested positive for glycophorin A. As a result, it was concluded that the figurine contained human peripheral blood but no menstrual fluid. The PMB test showed reliable results in a swab taken from a stain that was suspected to contain human blood of unknown age and time of deposition. A clear result was obtained confirming the suitability of this test for forensic settings.

### Sexual assault cases

All samples except the dress from case #1 reacted positive in the Feca-Cult test. These results were confirmed by the SERATEC PMB test. From ten samples, six presented positive results for menstrual blood. The positive detection of D-dimer in case #7 is somewhat surprising and might be explained by residual menstrual fluid in the vagina, i.e., through intermenstrual bleeding [21]. For cases #4, #8, #9, and #10, the incident happened within 3 days after menses, with consistent results. In four cases (#1, #2, #5, #6), the test reacted positive for hemoglobin presence but negative for D-dimer presence indicating blood of a traumatic cause. The test performance seems promising. A summary of the results is shown in Table 1.

## Conclusion and general considerations

The SERATEC PMB test proved to be a helpful tool in interpreting samples containing human menstrual and/or peripheral blood. Test cassettes were easy to use, interpretation of results was found to be straightforward and unambiguous. No special training was needed to apply the test successfully. It was shown that tests with a cut-off of 400 ng/ml overall produced clearer results with a stronger signal compared to 300 ng/ml. Therefore, this cut-off was chosen for the commercially available product. The results of this study show that the test’s detection limit is 240 nl of menstrual fluid, proving the high sensitivity of the assay. Highly sensitive tests are especially important for forensic and historical samples, as typically, only limited material is available for testing. Slight intra-individual differences were observed in terms of intensity of the test result but no significant differences in line intensities were received when testing menstrual blood from different days of the menses. With an incubation time of 90 min, it was possible to analyze aged-dried samples, which is also crucial for crime scene samples, especially considering stored evidence from so-called cold cases.

Biological secretions, such as vaginal fluid, semen, saliva, and urine, might be part of a mixed sample on evidence in cases of alleged sexual assault. This enhances the results reported here, since all mixtures containing menstrual blood resulted in a positive signal for D-dimer presence and no false-positive results were obtained for other tested body fluids, proving its high specificity. The test is human-specific and did not react with animal blood. Even though rabbit blood yielded a positive signal for D-dimer, no signal for hemoglobin was visible. Since menstrual blood contains hemoglobin, both lines need to be visible for a positive test result.

In addition, even the blood of elderly people who may present FDP does not appear to influence the test. None of the samples from donors over the age of 60 generated a false-positive result for D-dimer presence. Even though a theoretical risk remains to obtain a false-positive result from peripheral blood of an elderly person, the relevance of such a finding is limited in casework; if a blood stain from an alleged sexual assault case does react positive to D-dimer presence but is known to originate from an elderly person, it should be interpreted as a false-positive reaction based on the fact that menstrual fluid cannot be expected to originate from women after menopause.

Possible limitations need to be taken into consideration when analyzing samples with the SERATEC PMB test. D-dimers are normally not detectable in blood from healthy individuals but D-dimer levels can be elevated in patients suffering from acute peripheral thromboembolisms, pregnancy, previous surgery, or active malignancy, which may lead to a false-positive test result [19, 22]. In unclear cases, a reference sample should therefore be tested to avoid misinterpretation of a positive test result for menstrual fluid. Moreover, D-dimer levels have shown to rise in postmortem samples [18], which was confirmed in our study with 70% of the postmortem blood samples giving a light to strong positive result for D-dimer presence. This should be kept in mind as a possible source of false positivity when postmortem samples need to be assessed. However, this limitation is of only limited relevance because most blood samples observed at crime scenes can be expected to originate from a living (and therefore actively bleeding) individual. Aside from these limitations, the test is simple to implement into forensic workflows and it is possible to use the remaining buffer-sample solution or the sample pad for subsequent DNA analysis. The performance is not user-dependent as it is standardized and no special training of the analyst is needed. All the above considerations had been useful for the interpretation of the ten sexual assault casework samples. It is worth mentioning that the peripheral blood marker gave consistent results with human blood tests already in use in the forensic field.

The SERATEC PMB test is the first commercially available example for a multiplexed immunochromatographic assay for forensic body fluid identification targeting two different body fluids in a single test. This, in combination with the possibility of performing standard DNA profiling from leftover buffer-sample solution, is an important characteristic of this test because it ensures minimal sample loss during body fluid testing.

## Electronic supplementary material

ESM 1 (PDF 114 kb).

ESM 2 (PDF 664 kb).
