# Supplementary material for: Forensic differentiation between peripheral and menstrual blood in cases of alleged sexual assault—validating an immunochromatographic multiplex assay for simultaneous detection of human hemoglobin and D-dimer
Source: Int J Legal Med. 2017 Oct 23;132(3):683–90. doi: 10.1007/s00414-017-1719-y (PMC5919992; doi:10.1007/s00414-017-1719-y)
Supplement: Supplementary file 1 [file 414_2017_1719_MOESM1_ESM.pdf]

Supplementary Material – Document 1

Brief history of each case and sample from "Materials and Methods - 2.5 Sexual assault cases"

Case #1 A girl (14 y.) claimed to have been raped. The material sent to forensics was the victim's dress and panty, containing bloodstains. The victim reported to be a virgin and medical examination revealed hymenal rupture.

Case #2 A girl (14 y.) was attacked by a stranger who allegedly raped her. A medical examination revealed internal lesions of the vagina. The panty containing not saturated hematoid spots were sent to forensics.

Case #3 A teenager (17 y.) was allegedly forced to vaginal and oral sex by two men. The victim's panty was sent to the forensic examination.

Case #4 A woman (40 y.) claimed to have been forced to have sex with a prospective employer during a job interview. Medical examination revealed no lesions, and a vaginal swab was sent to forensics.

Case #5 A woman (24 y.) claimed to have been raped and robbed. Medical examination revealed no lesions, and a vaginal swab was sent to forensics, maintained frozen before analysis.

Case #6 A girl (13 y.) claimed to have been raped after leaving school on the way home by two men. No vaginal lesions were reported, and a vaginal swab was sent to forensics, maintained frozen before analysis.

Case #7 A girl (14 y.) claimed to have been sexually assaulted by her partner. Medical examination revealed no vaginal lesion. A vaginal swab was sent to forensics and maintained frozen before lab analysis.

Case #8 A woman (18 y.) claimed to have been raped by an unknown male through threat by knife. Medical examination revealed no bruises or vaginal lesion. A vaginal swab was sent to forensics and maintained frozen before lab analysis.

Case #9 A woman (27 y.) reported genital pain when waking up after a drinking night. Medical examination revealed bruises on the right side of the *labia minora*, but no vaginal lesion. A vaginal swab was sent to forensics and maintained frozen before lab analysis.

Case #10 A girl (14 y.) was reportedly kidnapped and sexually assaulted. Medical examination revealed no vaginal lesion. A vaginal swab was sent to forensics and maintained frozen before lab analysis.
