# Supplementary material for: Forensic differentiation between peripheral and menstrual blood in cases of alleged sexual assault—validating an immunochromatographic multiplex assay for simultaneous detection of human hemoglobin and D-dimer
Source: Int J Legal Med. 2017 Oct 23;132(3):683–90. doi: 10.1007/s00414-017-1719-y (PMC5919992; doi:10.1007/s00414-017-1719-y)
Supplement: Supplementary file 2 [file 414_2017_1719_MOESM2_ESM.pdf]

**Supplementary Material – Document 2**

**Electropherograms from “Results and Discussion - 3.3 DNA analysis”**

The STR-profiles were generated from A) the sample well and B) the sample-buffer-solution. The swabs contained a mixture of menstrual fluid and semen, which is represented in the electropherograms with a female and a male component. No significant differences in peak height or quality of the profiles results from the two collection methods.

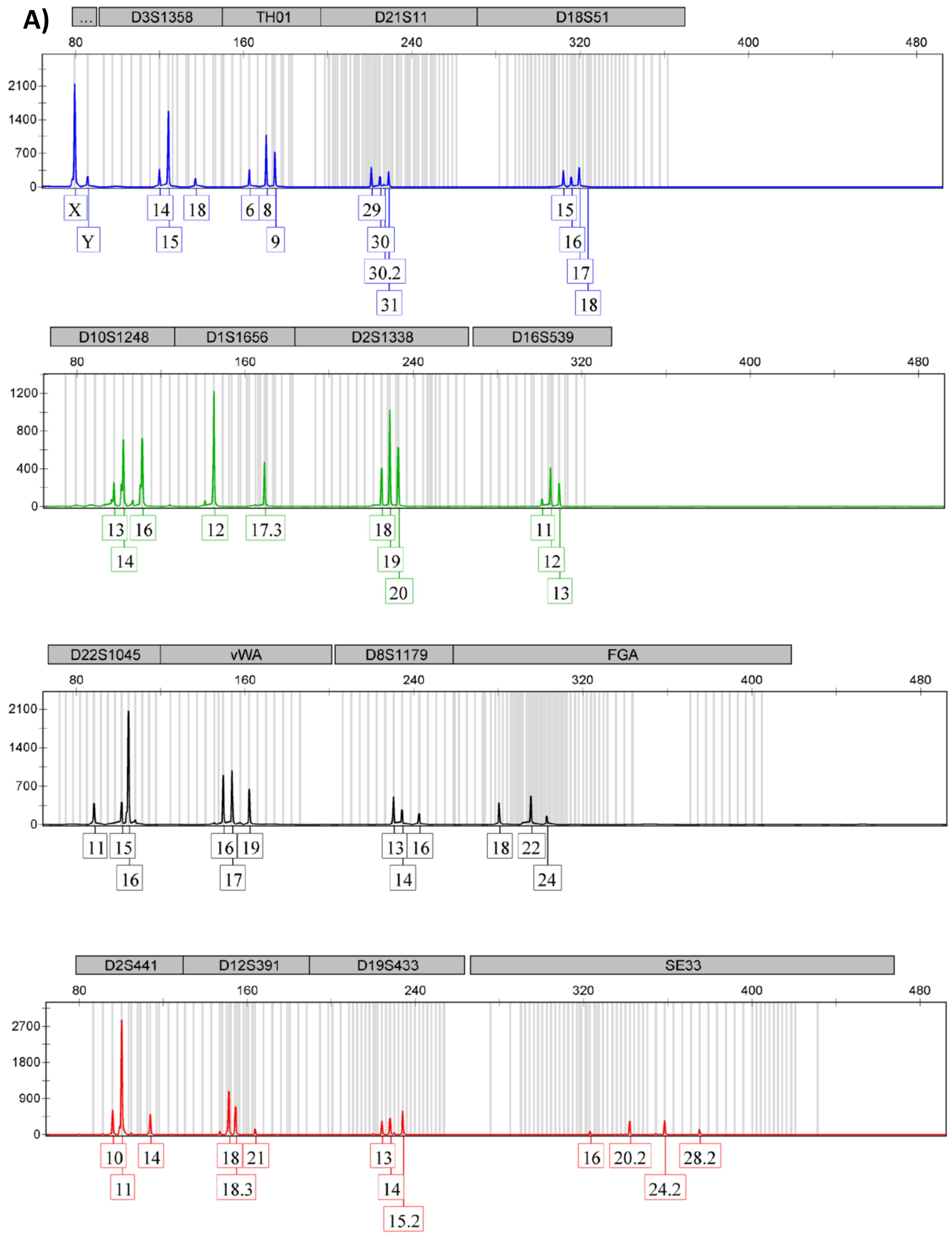

B)

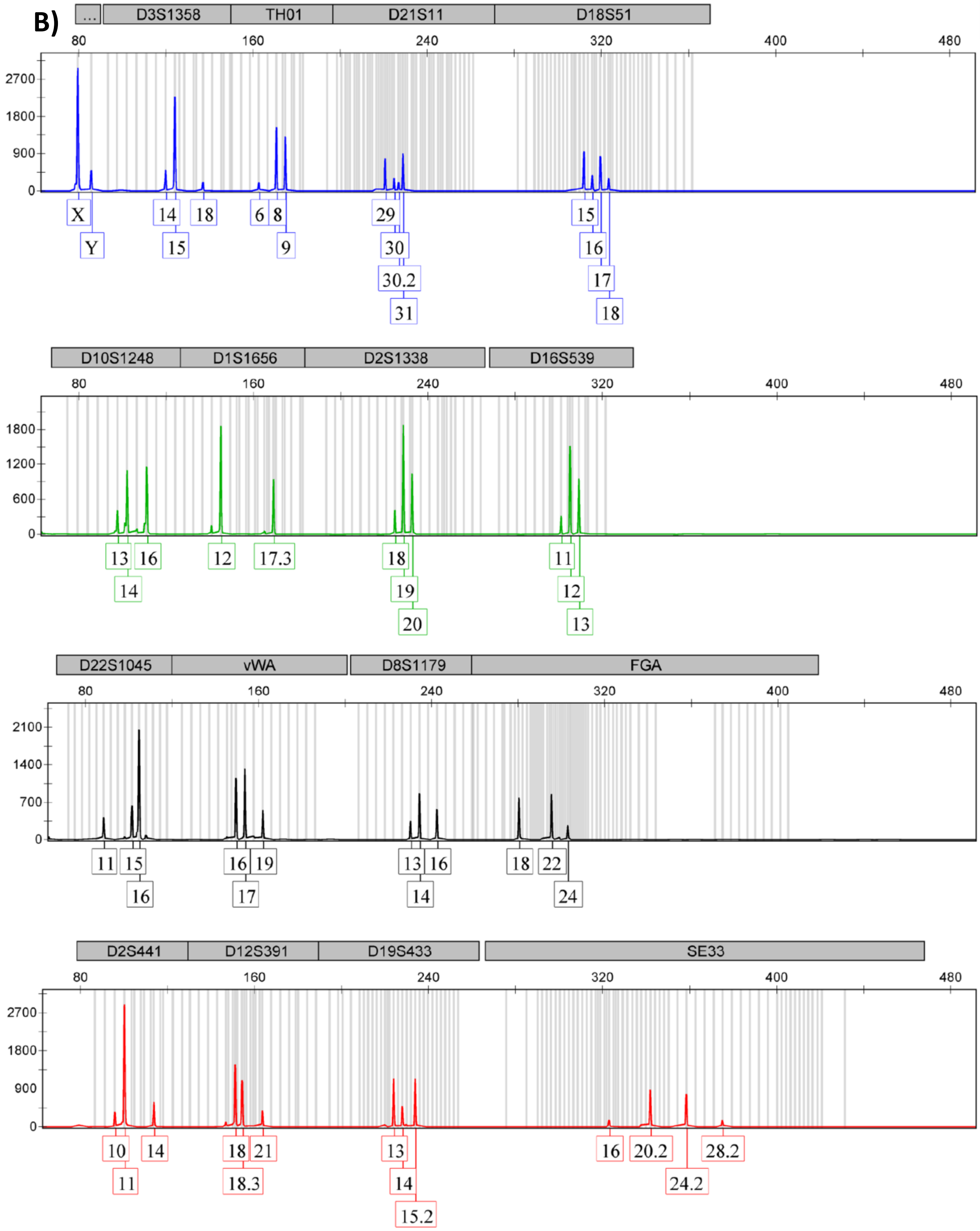
D3S1358
TH01
D21S11
D18S51
X
Y
14
15
18
6
8
9
29
30
30.2
31
15
16
17
18
D10S1248
D1S1656
D2S1338
D16S539
13
14
16
12
17.3
18
19
20
11
12
13
D22S1045
vWA
D8S1179
FGA
11
15
16
16
17
19
13
14
16
18
22
24
D2S441
D12S391
D19S433
SE33
10
11
14
18
18.3
21
13
14
15.2
16
20.2
24.2
28.2
